# The Role of Artificial Intelligence and Machine Learning Models in Antimicrobial Stewardship in Public Health: A Narrative Review

**DOI:** 10.3390/antibiotics14020134

**Published:** 2025-01-30

**Authors:** Flavia Pennisi, Antonio Pinto, Giovanni Emanuele Ricciardi, Carlo Signorelli, Vincenza Gianfredi

**Affiliations:** 1 Faculty of Medicine, University Vita-Salute San Raffaele, 20132 Milan, Italy; pennisi.flavia@hsr.it (F.P.); pinto.antonio@hsr.it (A.P.); ricciardi.giovanni@hsr.it (G.E.R.); signorelli.carlo@hsr.it (C.S.); 2 PhD National Program in One Health Approaches to Infectious Diseases and Life Science Research, Department of Public Health, Experimental and Forensic Medicine, University of Pavia, 27100 Pavia, Italy; 3 Department of Biomedical Sciences for Health, University of Milan, Via Pascal 36, 20133 Milan, Italy

**Keywords:** antimicrobial resistance, antimicrobial stewardship, artificial intelligence, machine learning, personalized antibiograms, diagnostic innovation, predictive models, public health

## Abstract

Antimicrobial resistance (AMR) poses a critical global health threat, necessitating innovative approaches in antimicrobial stewardship (AMS). Artificial intelligence (AI) and machine learning (ML) have emerged as transformative tools in this domain, enabling data-driven interventions to optimize antibiotic use and combat resistance. This comprehensive review explores the multifaceted role of AI and ML models in enhancing antimicrobial stewardship efforts across healthcare systems. AI-powered predictive analytics can identify patterns of resistance, forecast outbreaks, and guide personalized antibiotic therapies by leveraging large-scale clinical and epidemiological data. ML algorithms facilitate rapid pathogen identification, resistance profiling, and real-time monitoring, enabling precise decision making. These technologies also support the development of advanced diagnostic tools, reducing the reliance on broad-spectrum antibiotics and fostering timely, targeted treatments. In public health, AI-driven surveillance systems improve the detection of AMR trends and enhance global monitoring capabilities. By integrating diverse data sources—such as electronic health records, laboratory results, and environmental data—ML models provide actionable insights to policymakers, healthcare providers, and public health officials. Additionally, AI applications in antimicrobial stewardship programs (ASPs) promote adherence to prescribing guidelines, evaluate intervention outcomes, and optimize resource allocation. Despite these advancements, challenges such as data quality, algorithm transparency, and ethical considerations must be addressed to maximize the potential of AI and ML in this field. Future research should focus on developing interpretable models and fostering interdisciplinary collaborations to ensure the equitable and sustainable integration of AI into antimicrobial stewardship initiatives.

## 1. Introduction

Antimicrobial resistance (AMR) is one of the most significant public health challenges of the 21st century. Recent studies estimate that AMR could result in up to 10 million deaths annually by 2050, posing a substantial threat to global healthcare systems and economies [1]. The World Health Organization (WHO) has declared AMR a critical priority, urging nations to implement innovative antimicrobial stewardship (AMS) strategies to curb the emergence of multidrug-resistant organisms (MDROs) [2].

Traditional antibiotics often fail to address infections caused by resistant pathogens, resulting in increased morbidity, mortality, and healthcare costs [3]. Additionally, the antibiotic development pipeline faces critical hurdles, including high costs, lengthy timelines, and low economic incentives for pharmaceutical companies, leading to stagnation in the production of novel agents [4,5]. Moreover, traditional AMS practices have relied on culture-based diagnostics, which often require 24 to 72 h to yield results. This delay compels clinicians to initiate empiric therapy with broad-spectrum antibiotics, which, while effective in covering likely pathogens, significantly contributes to the selection pressure driving resistance [6]. Furthermore, the limitations of the existing antibiotics are further compounded by their broad-spectrum nature, which disrupts normal microbiota and increases the risk of secondary infections, such as Clostridioides difficile [7,8]. The global disparity in access to effective antibiotics, particularly in low- and middle-income countries, exacerbates the AMR crisis by fostering the spread of resistance through suboptimal treatments. Additionally, Logistic Regression (LR) and other classical statistical methods have served as foundational tools for predicting AMR. However, these methods often struggle to analyze the non-linear relationships and large-scale datasets that characterize modern healthcare environments [9]. These challenges underscore the urgent need for innovative approaches, such as leveraging artificial intelligence (AI) and machine learning (ML), to optimize antibiotic use and improve stewardship practices.

AI and ML offer transformative solutions to overcome these limitations, enabling real-time data-driven interventions, including complex datasets, microbiological data, and genomic information analysis to enhance AMS programs globally. By leveraging electronic health records (EHRs), microbiological data, and patient demographics, ML algorithms can identify resistance patterns, predict susceptibility, and guide antibiotic selection with unprecedented precision [10].

Prominent applications of ML in AMS include predicting resistance to specific antibiotics, optimizing empiric therapy, and integrating patient-specific factors to develop personalized antibiograms. For instance, a study utilizing gradient-boosted trees demonstrated AUC values exceeding 0.87 in predicting resistance patterns across multiple bacterial species [11]. Furthermore, neural networks have been applied to rapidly analyze genomic data, identifying resistance determinants in hours rather than days. Moreover, AI has transformative implications for public health by enabling real-time surveillance, the early detection of emerging threats, and targeted interventions, which enhance the efficiency and effectiveness of responses to antimicrobial resistance and infectious diseases. 

Globally, regulatory agencies, including the Food and Drug Administration (FDA) and the European Medicines Agency (EMA), have begun exploring the integration of ML-based technologies into healthcare [12,13], spanning applications such as medical imaging [14], disease diagnosis [15], and patient management [16].

However, several challenges hinder the full implementation of AI in AMS. Data standardization across healthcare systems remains a significant obstacle, with inconsistencies in EHR formats complicating model training. Additionally, ethical considerations surrounding algorithm transparency, data privacy, and equitable access to AI technologies must be addressed to maximize their potential [17].

This comprehensive review examines the transformative role of AI and ML in AMS, highlighting their applications in resistance prediction, diagnostic innovation, and the development of personalized antibiograms. In detail, this review aims to (1) synthesize evidence on the applications of AI and ML in AMS, with a focus on resistance prediction, diagnostic advancements, and public health strategies; (2) identify gaps in the current research and implementation, such as issues related to model transparency, ethical considerations, and scalability; and (3) propose future directions for the integration of AI/ML technologies into AMS, emphasizing their role in addressing global public health priorities.

By exploring these objectives, this article aims to underscore how these AI-driven solutions can address the global AMR crisis by providing actionable insights for researchers, clinicians, and policymakers, with the final aim of advancing the development of sustainable and equitable strategies.

## 2. AI and ML Models in Antimicrobial Stewardship: From Predictive Models to Clinical Impact

**Figure 1:**
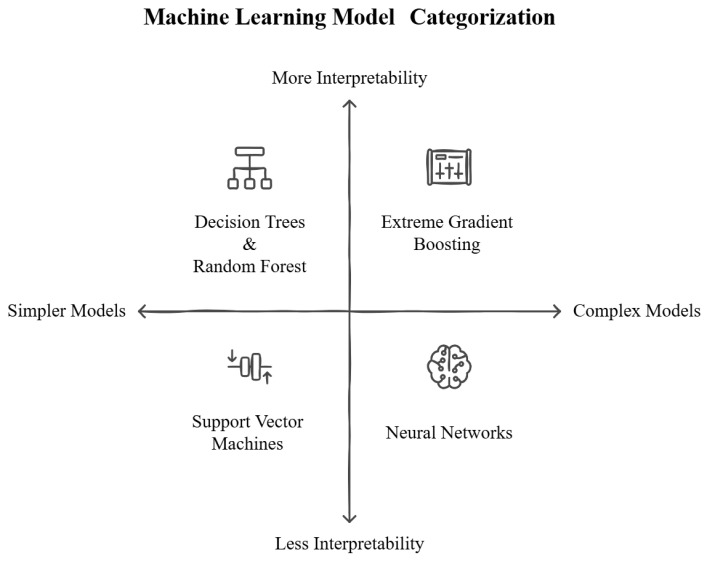
Categorization of machine learning models by complexity and interpretability. Models like decision trees and random forests prioritize simplicity and interpretability, while neural networks offer higher complexity and lower interpretability. Support Vector Machines and Extreme Gradient Boosting occupy intermediate positions, balancing these characteristics.

Since AMS is one of the main challenges of public health, several efforts have been put in place; among them, the last frontier is machine learning algorithms. Prominent ML models (Figure 1) employed in AMS include the following:

- Decision Trees (DTs) and Random Forests (RFs): These models are valued for their interpretability and ability to handle both categorical and numerical data. They excel at identifying clear patterns within heterogeneous datasets, such as those derived from EHRs.
- Boosted Models: Examples include Extreme Gradient Boosting (XGBoost), which combines the outputs of multiple decision trees to enhance predictive accuracy in complex datasets.
- Support Vector Machines (SVMs): These models are effective in datasets with a high number of variables, although their scalability is limited for large datasets.
- Neural Networks (NNs): Particularly recurrent neural networks, which are well suited for analyzing intricate data and temporal sequences, such as genomic information. While these models are less interpretable, they are highly effective for processing large-scale, high-dimensional datasets.

These advanced ML models have significantly enhanced the ability to predict antimicrobial resistance, optimize empiric antibiotic selection, and minimize the risk of therapeutic failure. By processing interconnected variables and incomplete datasets, they generate actionable insights that exceed the capabilities of traditional approaches. Traditionally, AMS relied on classical statistical methods, such as LR, to support therapeutic decision making. While these models are simple and interpretable, they have shown substantial limitations in handling the increasing complexity of clinical and microbiological data. LR, for instance, was effective in estimating the probability of resistant infections based on a limited set of well-defined variables, such as prior antibiotic use or the presence of comorbidities [18,19]. Nevertheless, despite the fact that ML models have demonstrated improved predictive capabilities, they also face notable limitations. Neural networks, for instance, often operate as “black-box” models, making their decision-making processes difficult for clinicians to interpret [20,21]. This lack of transparency can hinder trust and adoption in clinical workflows. Random forests and XGBoost, although interpretable compared to neural networks, may require extensive computational resources and are prone to overfitting in small or imbalanced datasets. Moreover, model performance is highly dependent on the quality and representativeness of training data, with biases in datasets potentially leading to inaccurate resistance predictions or inequitable outcomes [22,23].

A recent meta-analysis, including 80 studies, demonstrated that ML models exhibited strong predictive performance and diagnostic accuracy, with the following results: AUC [ES: 72.28 (70.42–74.14)], accuracy [ES: 74.97 (73.35–76.58)], sensitivity [ES: 76.89; (71.90–81.89)], specificity [ES: 73.77; (67.87–79.67)], NPV [ES:79.92 (76.54–83.31)], and PPV [ES: 69.41 (60.19–78.63)] across various AMS settings [24].

A study developed machine learning models to predict the incidence of carbapenem-resistant Gram-negative bacteria (CR-GNB) within a week, using data from 2015 to 2019. RF has outperformed the multivariate LR in terms of accuracy (84% vs. 72%) and AUC (0.91 vs. 0.78). These ML-based models enable the real-time identification of high-risk patients in intensive care settings, offering improved predictive performance over traditional methods [25]. A USA study has introduced PyTorch_EHR, a deep learning model that uses EHR time series data to predict the positivity of methicillin-resistant staphylococcus aureus culture (MRSA) within two weeks. Using data from 8164 MRSA and 22,393 non-MRAA events, PyTorch_EHR passed the LR (AUC: 0.911 vs. 0.857). External validation with the MIMIC-IV dataset confirmed its superior accuracy (AUC: 0.859 vs. 0.816), demonstrating its potential for better stratification of MRSA risk [26].

A Chinese manuscript developed risk prediction models for acute kidney injury (AKI) in severely burned patients comparing LR to Extreme Gradient Boosting (XGBoost) machine learning. Data from 157 patients showed that XGBoost outperformed LR with a higher AUC (0.920 vs. 0.875), specificity (89.7% vs. 84.4%), and sensitivity (82.0% vs. 77.7%). The key predictive variables included APACHE II (Acute Physiology and Chronic Health Evaluation II) score, sepsis, fluid resuscitation, and burn severity. ML demonstrated superior accuracy and potential for clinical application in individualized AKI prediction than LR [27]. For example, ML models have been employed to predict multidrug resistance in patients with severe infections, reducing response times and improving clinical outcomes. Specifically, these models identified more targeted antibiotics with higher efficacy compared to broad-spectrum agents, leading to shorter hospital stays and fewer secondary infections, such as *Clostridioides difficile* [28,29]. ML models have also been developed in the medical field to assess the adequacy of antimicrobial surgical prophylaxis, addressing the inefficiency of manual reviews. Using a dataset of 601 instances with 26 attributes, algorithms such as multilayer perceptron, SimpleLogistic, and DT achieved outstanding performance (AUC > 0.97). Bagging (Bootstrap Aggregating) and SMOTE (Synthetic Minority Oversampling Technique) have improved predictions on unbalanced datasets. These models can support antimicrobial management teams in optimizing surgical prophylaxis [30]. A key data source leveraged by ML models is EHRs, which integrate comprehensive patient information, including medical histories, laboratory results, medication regimens, and vital signs. By utilizing EHR data, ML algorithms can manage vast arrays of clinical variables, delivering faster and more precise resistance predictions [31,32].

Another study, conducted in the Netherlands, demonstrated how ML algorithms, specifically SVM, can outperform traditional methods, such as LR, in managing urinary tract infections (UTIs). The researchers developed a decision support system to predict UTIs by integrating data from urinalysis and culture results, achieving significant outcomes. SVM achieved an AUC of 80.43 ± 0.95, surpassing LR, which was limited to 69.86 ± 1.81, indicating superior ability in distinguishing infections from non-infections. Furthermore, with additional improvements using the semi-supervised RESSEL (Reliable Semi-Supervised Ensemble Learning) method, RF increased its accuracy to 76.77 ± 0.97, while SVM maintained a competitive AUC of 78.20 ± 0.96 [33].

Research investigates the potential of the use of the NN and LR methods in the diagnosis of methicillin-resistant *Staphylococcus aureus* (MRSA). It was found that NN is better than the LR, both in terms of discriminating power and robustness. With the modeling flexibility inherent in its techniques, NN is effective in dealing with MRSA and other classification problems involving a large number of variables and interaction complexities. On the other hand, the LR is slightly lower, although it offers more clarity and less perplexity. The latter may, therefore, be a method of choice when fewer variables are involved and/or results are to be justified [34]. However, as healthcare systems generated increasingly large and heterogeneous datasets, these traditional methods struggled to capture non-linear relationships and adequately process such complexity [35]. The introduction of ML models has marked a turning point in AMS, offering tools capable of rapidly processing large datasets and identifying intricate patterns that would be imperceptible with conventional methods. A study evaluated the use of the NN and ML algorithms to predict extended-spectrum beta-lactamase production (ESBL) in Enterobacteriaceae causing community-onset bacteremia in a high-ESBL prevalence area, comparing them to conventional multivariable LR. Analyzing 5625 patients from three Hong Kong hospitals, NN achieved superior predictive performance (Area Under Curve AUC 0.761, F1 score 0.661) compared to LR (AUC 0.667, F1 score 0.596). NN showed higher specificity (91.5%) but lower sensitivity (37.5%), demonstrating its potential advantage in guiding empirical antimicrobial therapy [36].

Despite their potential, ML models face significant challenges in their implementation. Algorithm transparency remains a critical issue: clinicians require interpretable models to integrate these tools effectively into clinical workflows. Integration into clinical workflows also poses logistical challenges. ML algorithms must be coupled with intuitive user interfaces and seamlessly embedded into the existing decision-making systems. Interdisciplinary collaboration among microbiologists, clinicians, and data scientists is essential to overcome these barriers and maximize the clinical impact of ML in AMS [37,38].

## 3. Revolutionizing Diagnostics: AI-Driven Rapid Pathogen Identification and Personalized Antibiogram

**Figure 2:**
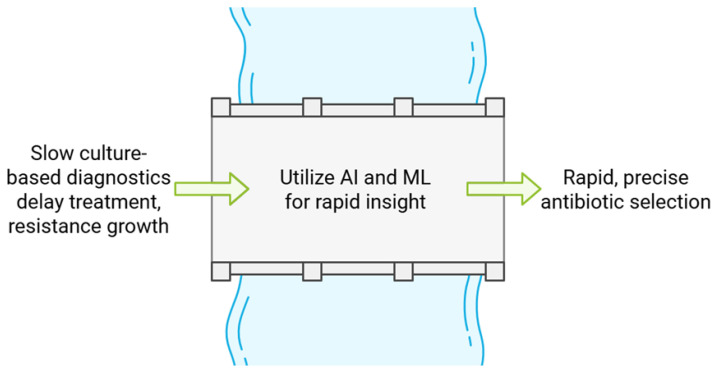
How artificial intelligence and machine learning improve antimicrobial stewardship: overcoming the ‘river’ of obstacles of traditional diagnostics to achieve rapid and accurate antibiotic selection.

Diagnostics play a pivotal role in AMS by informing the selection of appropriate antibiotics. However, traditional diagnostic methods, such as culture-based susceptibility testing, have inherent limitations. These include extended processing times (24–72 h), which often delay the initiation of targeted therapy, and reduced utility in resource-limited settings [39]. In this context, clinicians frequently resort to empiric antibiotic therapy, relying on broad-spectrum agents to ensure coverage while awaiting diagnostic results. While this approach addresses immediate clinical needs, it risks exacerbating AMR and harming patient microbiota [40]. The emergence of AI and ML has transformed this landscape, enabling rapid pathogen identification and the development of personalized antibiograms, which significantly enhance diagnostic precision and therapeutic efficacy (Figure 2).

AI models have demonstrated exceptional capabilities in pathogen identification by analyzing genomic, proteomic, and phenotypic data. Mass spectrometry data, for instance, when combined with ML algorithms such as gradient-boosted trees, random forest, or SVM, has been used to identify carbapenem-resistant *Klebsiella pneumoniae* with AUC values exceeding 0.85, providing actionable results within hours compared to the days required for conventional diagnostics [41,42]. This rapid turnaround time allows for more targeted therapeutic decisions, reducing the reliance on empiric broad-spectrum antibiotics. Similarly, neural networks have been applied to electronic medical records, enabling the rapid differentiation of bacterial species and the prediction of their resistance profiles [43]. Natural language processing models have also been integrated into diagnostic workflows to enhance the interpretation of microbiological reports, linking them with patient histories to refine treatment recommendations. These advancements have drastically reduced diagnostic turnaround times, with some systems providing actionable results within hours compared to the days required for traditional culture-based methods [44]. However, these methods are not without challenges. False positives and negatives remain a significant concern, particularly in resource-constrained settings where validation with advanced laboratory tools is unavailable. For example, a high rate of false negatives in resistance profiling could lead to under-treatment, while false positives may drive unnecessary broad-spectrum antibiotic use. Additionally, many AI-based diagnostic tools rely on proprietary algorithms, raising concerns about reproducibility and transparency.

Traditional antibiograms, which aggregate resistance data from previous cases, have been invaluable tools for guiding empiric therapy. However, they present several limitations:

- Generalization: they provide population-level insights rather than patient-specific guidance;
- Time lag: resistance patterns may evolve faster than antibiogram updates;
- Resource dependence: in resource-limited settings, antibiograms may be incomplete or unavailable.

ML has addressed these issues through the creation of personalized antibiograms, which integrate patient-specific factors—such as previous antibiotic exposure, comorbidities, and local resistance patterns—with real-time microbiological data. A study by Tzelves et al. demonstrated that ML-driven antibiograms reduced broad-spectrum antibiotic use while maintaining equivalent coverage rates, particularly in urinary tract infections [45]. This approach minimized the risk of resistance development and improved the precision of empiric therapy. A Turkish study investigated the use of machine learning to assess antibiotic resistance in *Escherichia coli* without relying on traditional antibiogram methods. Models such as k-Nearest Neighbors, Artificial Neural Networks, SVM, and DT were employed to analyze clinical data from 103 patients diagnosed with *E. coli* infections. The results demonstrated high accuracy rates in predicting sensitivity/resistance for antibiotics such as fosfomycin (98%), levofloxacin (98%), and ertapenem (96%), highlighting the potential of AI-driven approaches to reduce time and costs in laboratory medicine decision-making processes [46]. A Spanish study aimed to predict antibiogram results for multidrug-resistant bacteria in intensive care unit (ICU) patients using machine learning techniques. Clinical variables from EHR were utilized, including individual antibiotic consumption and medications administered to other ICU patients. Data from 3476 ICU admissions at Fuenlabrada University Hospital (Madrid) between 2004 and 2020, including 628 multidrug-resistant cases, were analyzed. The highest accuracy (77%) and specificity (82%) were achieved with random forest classifiers, while the highest sensitivity (69%) and ROC-AUC (76%) were obtained using Chi-square feature selection and LR or XGBoost classifiers [47]. A multi-site retrospective study in the USA developed machine learning models to predict personalized antibiograms using EHR from 8342 infections at Stanford emergency departments and 15,806 uncomplicated urinary tract infections from Massachusetts General Hospital and Brigham & Women’s Hospital in Boston. Personalized antibiograms demonstrated comparable or improved antibiotic coverage rates compared to clinicians (85.9% vs. 84.3% at Stanford, *p* = 0.11; 90.4% vs. 88.1% in Boston, *p* < 0.0001). They achieved similar coverage while significantly narrowing broad-spectrum prescriptions, such as reducing vancomycin + piperacillin/tazobactam use by 69% at Stanford. These findings suggest that precision empiric antibiotic prescriptions using personalized antibiograms can enhance patient safety and antibiotic stewardship by minimizing unnecessary broad-spectrum antibiotic use and combating antimicrobial resistance [48]. Furthermore, these models allow clinicians to tailor empiric therapy with a higher likelihood of success, reducing the duration of hospital stays and the risk of complications such as *Clostridioides difficile* infections [49].

Therefore, the integration of AI and ML into AMS has revolutionized diagnostic and therapeutic processes, allowing for rapid, accurate, and customized approaches to antibiotic selection. By addressing the limitations of traditional methods, these advances not only optimize patient outcomes but also play a key role in mitigating the global challenge of AMR.

## 4. AI-Enhanced Public Health Strategies for AMR Control

AI is profoundly reshaping public health strategies to address AMR, a major global health threat that endangers the effectiveness of infectious disease treatments and the resilience of health systems. Declared one of the top ten public health challenges by the WHO [2], AMR is threatening decades of medical progress, making coordinated and innovative public health interventions essential. AI has emerged as a cornerstone in this effort, offering unparalleled capabilities to optimize AMS programs, enhance surveillance, and strengthen global health infrastructure.

ML applications in healthcare have primarily focused on predictive tasks across various clinical domains. In public health contexts, performance metrics provide insight into how well models can identify at-risk populations and inform intervention strategies. For instance, Ghosh et al. employed ensemble ML models to predict susceptibility phenotypes across common pathogen–antibiotic combinations in septic ICU patients, offering tools to optimize treatment and reduce resistance spread [50]. Similarly, Feretzakis et al. demonstrated the potential of ML to predict AMR in a single-center ICU study in Greece [51], while Goodman et al. applied an ML-derived decision tree to identify bloodstream infections caused by extended-spectrum beta-lactamase-producing organisms [52]. While these studies highlighted the potential of ML in AMR management through AUROC-based evaluations, they did not compare model performance against clinicians using real-world, unseen data. This gap underscores the need for public health-focused assessments to evaluate how ML tools can complement or enhance clinical workflows, ensuring their effectiveness and reliability in real-world applications. In community and resource-limited settings, ML applications are beginning to show potential as well. For example, Oonsivilai et al. compared eight ML models for predicting resistance in Cambodian children with bacteremia, achieving AUROCs between 0.7 and 0.8 [53]. Yelin et al. developed an ML-based clinical decision support system (CDSS) for UTIs, leveraging insurance claims, susceptibility data, and limited clinical metadata to reduce inappropriate antibiotic prescriptions by 30% [54]. Similarly, Kanjilal et al. focused on using ML to predict resistance in outpatients with uncomplicated UTIs, highlighting its role in less acute but still high-priority public health contexts [55]. Studies addressing ML applications in low-income and high-risk populations remain scarce. Future efforts must prioritize designing ML applications for resource-constrained environments, where AMR poses a disproportionate burden. Addressing these gaps is essential for ensuring that ML-driven public health interventions are both equitable and globally impactful, fostering better AMR management across diverse healthcare settings, and also among low- and middle-income countries (LMICs) that often bear a disproportionate burden of AMR, with widespread empirical use of broad-spectrum antibiotics further accelerating resistance. ML models could play a transformative role in these settings by enabling cost-effective and scalable solutions, such as predictive algorithms for resistance patterns and optimized antibiotic recommendations based on minimal clinical data.

In high-income countries, ML could target high-risk demographics, including immunocompromised patients, individuals undergoing chemotherapy, and residents in long-term care facilities, who are particularly vulnerable to multidrug-resistant infections [56]. For example, personalized antibiograms powered by ML could support clinicians in tailoring empiric therapy, reducing reliance on broad-spectrum antibiotics and improving clinical outcomes [57].

Additionally, ML models have the potential to benefit pediatric and geriatric populations, where antibiotic selection must be carefully calibrated to account for physiological differences and susceptibility to adverse drug effects. Integrating ML into AMS initiatives in these demographics could enhance therapeutic precision while minimizing risks [58].

Lastly, ML-based surveillance systems could address geographic disparities in AMR monitoring by providing real-time insights into resistance trends, even in resource-constrained environments [59]. Such systems could guide targeted public health interventions, ensuring that resources are directed to regions with the greatest need.

However, achieving equity requires overcoming barriers such as the digital divide, ensuring affordability, and fostering local capacity for the development and maintenance of AI systems. Collaborative efforts between global health organizations, governments, and technology developers are essential to scale these solutions sustainably.

By leveraging diverse and integrated datasets, including EHRs, laboratory results, environmental monitoring data, and genomic information, AI-driven tools enable public health systems to predict, prevent, and mitigate resistance with unprecedented speed and accuracy [43,48,53,54,60,61]. EHRs are an invaluable resource for public health efforts to combat AMR, as they contain critical data that are routinely used to guide therapeutic decisions. This includes essential information such as (1) patient history of colonization or infection with multidrug-resistant organisms; (2) susceptibility test results; (3) signs and symptoms with their progression over time and response to previous treatments; (4) laboratory test results, including acute phase markers and organ dysfunction indicators; (5) baseline comorbidities; (6) concurrent infectious and non-infectious acute-phase conditions; (7) reported allergies; and (8) concurrent antimicrobial and non-antimicrobial treatments [62]. Manually synthesizing these data for each patient requires considerable time and effort, often taking several minutes per consultation. ML models, however, can analyze the same data and generate predictions in seconds or less, making these tools a transformative asset in public health and clinical decision making. The integration of reliable ML-based clinical decision support systems (ML-CDSSs) has the potential to significantly reduce the burden on healthcare professionals, particularly in antimicrobial stewardship programs [62]. By automating the review and analysis of data, ML-CDSSs free experts to focus on higher priority public health tasks, such as making complex diagnostic and therapeutic decisions, optimizing antimicrobial use, and addressing broader health system needs. This efficiency is particularly important in resource-constrained settings, where public health and clinical professionals are often overwhelmed by competing demands. Furthermore, by streamlining the decision-making process, ML-CDSSs can improve the overall effectiveness of antimicrobial stewardship initiatives, thereby enhancing the ability of health systems to combat AMR and safeguard public health outcomes. However, using EHR data for ML-based CDSS presents several challenges [63]. It is often prone to errors or indecipherable entries due to data entry issues. The complexity of AMR requires integrating data from multiple sources, making validation and cleaning processes time-intensive [64]. Additionally, EHR data may suffer from non-random missing observations, confounding by indication, and selection bias [65]. Susceptibility data can be affected by evolving clinical breakpoints and shifting distributions over time, potentially reducing model accuracy when trained on historical data. Furthermore, data representations are often institution-specific, limiting generalizability [66]. Addressing these issues is essential to ensure that ML CDSS provides accurate, locally relevant recommendations.

AI has demonstrated significant potential in both resource allocation and surveillance enhancement, addressing critical challenges in public health. By analyzing historical trends, antibiotic usage patterns, and environmental factors, AI models can identify high-risk zones where resistance is likely to surge. During the COVID-19 pandemic, AI demonstrated its utility in optimizing resource use by integrating AMR and viral surveillance data to identify and mitigate antibiotic overuse, supporting rapid policy adaptation. Such insights allowed health systems to adapt policies rapidly, highlighting AI’s potential to support evidence-based decision making in dynamic and resource-constrained scenarios [67,68,69,70]. Such applications underscore AI’s role in evidence-based decision making, especially in dynamic and resource-constrained scenarios. For example, predictive tools like those developed by Metabiota alerted several countries, including Japan, Thailand, Taiwan, and South Korea, to the coronavirus outbreak a week before official announcements, allowing these nations to prepare and allocate resources proactively [71]. Wieczorek et al. enhanced their model with adaptive techniques to adjust automatically to real-time data and patterns, achieving an overall prediction accuracy of 88% and up to 99% in specific regions [72]. However, the generalizability of these models across diverse geographic and socio-economic settings is often limited. For instance, models trained on data from high-resource healthcare systems may perform poorly in low-resource environments due to differences in data availability, reporting standards, and local resistance patterns. Additionally, operational challenges such as inconsistent data entry in electronic health records and a lack of interoperability across systems may reduce the reliability of these tools in real-world public health applications.

AI’s role in public health extends to global coordination, facilitating international efforts to combat AMR. By linking local surveillance data to global networks like the WHO’s Global Antimicrobial Resistance and Use Surveillance System (GLASS) [73], AI enhances cross-border monitoring and response capabilities. This global perspective is essential for addressing AMR, a threat that transcends national boundaries. AI-driven insights can inform the development of international guidelines, harmonize reporting standards, and foster collaborative research, all of which are critical for a unified global response to AMR. Furthermore, AI aids in evaluating the impact of interventions, enabling the continuous improvement of public health strategies.

Despite its transformative potential, the integration of AI into public health systems faces relevant challenges. Ensuring the quality, consistency, and interoperability of data across diverse health systems is paramount [74]. Standardizing data collection protocols and improving algorithm transparency are essential to building trust among public health stakeholders, including healthcare providers, policymakers, and communities [75,76]. Additionally, the presence of publication bias in some studies highlights the need for the rigorous, independent validation of AI models to ensure their reliability and generalizability [77]. Addressing these challenges is critical to unlocking the full potential of AI in AMR management.

In conclusion, AI represents a paradigm shift in public health approaches to AMR control. By enhancing diagnostic precision, enabling more effective resource allocation, and fostering international collaboration, AI aligns with the objectives of the WHO’s Global Action Plan [78] to combat AMR. These objectives include raising awareness, improving surveillance, optimizing antimicrobial use, and fostering sustainable investments in innovative solutions. Through its capacity to deliver scalable, data-driven, and actionable insights, AI offers public health systems the tools to address AMR comprehensively, safeguarding the effectiveness of antimicrobial treatments and promoting the One Health vision of interconnected human, animal, and environmental health. By addressing the existing barriers and fostering global cooperation, AI-driven public health strategies hold the promise of transforming AMR control into a sustainable and impactful global health priority.

## 5. Overcoming Barriers to AI Implementation in AMS

Implementing AI and ML models in AMS presents several complex challenges that can hinder their effective use in improving public health. These challenges can be divided into three main categories: ethical, technical, and operational.

Ethics has become an increasingly vital focus in the development and implementation of artificial intelligence across a wide range of sectors, particularly in healthcare. As AI technology rapidly evolves and integrates into critical areas of society, the potential for ethical dilemmas and unintended consequences has prompted a proactive response. In light of these challenges, a variety of initiatives have been launched, including the formation of specialized organizations [79,80,81] and the creation of comprehensive principle documents [82,83,84]. These resources are thoughtfully designed to guide AI practitioners and establish best practices, ensuring that ethical considerations are at the forefront of AI’s advancement and deployment.

Data privacy management is one of the most critical issues. AI and ML inherently depend on extensive datasets for effective training and model improvement. This reliance on large volumes of data introduces significant concerns regarding the privacy of sensitive patient information. Such data may include critical aspects like disease risks, personal lifestyle choices, mental health conditions, family dynamics, and sexual orientation [85,86,87]. The potential for this information to be collected, utilized, and possibly disclosed for non-medical purposes raises alarming questions about privacy violations and the ethical implications of managing personal health data. There is a legitimate concern regarding potential discrimination based on an individual’s health status or future health risks [88,89]. Sharing data with third parties carries the risk of making individuals susceptible to data misuse. This can occur through cyber-theft or even accidental leaks of information [90].

Bias in AI models is a critical ethical issue. If training data does not represent the diversity of the population, the resulting models may perform unevenly across different demographic groups. This can lead to clinical decisions that reinforce healthcare disparities and undermine equitable treatment [91,92,93]. For instance, if an algorithm is developed using data primarily from a demographic where a particular disease is more prevalent, it is likely to erroneously associate that disease with that specific ethnicity. This flawed association can result in misdiagnosis and inappropriate treatment for individuals from other ethnic backgrounds, further entrenching health inequities [94,95]. Bias can also be introduced during the algorithm design phase. The choices of developers regarding variables to include, metrics to use, and parameters to set can influence the behavior of the algorithm and introduce bias [96].

Technical challenges are significant, particularly data quality, which hinders the effective use of AI and ML in AMS. The successful training of machine learning models requires large amounts of accurate and representative data. However, the available training data are often incomplete, non-standardized, or of poor quality [97]. For example, research has revealed that microbiological data can exhibit significant variability across different institutions [98]. This inconsistency presents a considerable challenge to the development of models that can be generalized across various settings. As a result, the ability to create reliable, effective AI and ML applications in AMS is hindered.

A major technical challenge is validating AI models in real clinical environments to ensure their predictions are accurate and actionable in daily medical practice. Many prototypes have been developed in controlled settings, but their real-world effectiveness remains unproven. There is a pressing need for more randomized clinical trials to assess AI tools’ performance in varied medical scenarios [99]. These trials will determine if these technologies can provide benefits in real patient care, complicated by the need to customize AI models for local populations and healthcare contexts. This necessity adds complexity to the validation process, highlighting the importance of a flexible approach to integrating AI into clinical practice.

Implementing AI in AMS programs faces several operational challenges, such as inadequate organizational support, limited resources, insufficient staff training, and difficulties integrating new technologies. A key obstacle is a lack of backing from hospital leadership, as research indicates that strong management commitment is crucial for the successful implementation of AMS programs. For example, a study conducted in hospitals in the Latin American region revealed that formal leadership support was often limited, leading to the poor implementation of AMS activities [100]. Without a clear mandate and allocated resources, programs can be perceived as a secondary initiative rather than a strategic priority.

Incorporating AI solutions into healthcare facilities’ established workflows presents a noteworthy challenge. For AI technologies to function effectively, they must seamlessly integrate with the existing information systems, particularly electronic health records (EHRs). Unfortunately, numerous healthcare institutions struggle with this integration process, often due to outdated technological infrastructure that cannot support the demands of modern AI tools [101]. Moreover, most studies utilized data derived from EHRs, a source that, while rich in clinical information, is often subject to institution-specific practices and biases. These biases can arise from variations in documentation standards, healthcare delivery models, and patient populations served by different institutions. Such factors may limit the generalizability of our findings to broader, more heterogeneous populations.

Additionally, the non-standardized nature of EHR data can introduce challenges, including variability in the granularity and accuracy of the recorded information. For instance, inconsistencies in coding practices or missing data elements may affect the performance of the AI and ML models developed using EHR data. These issues are well documented in the literature and underscore the importance of data standardization for improving the external validity of research findings.

To mitigate these limitations, future studies could leverage multi-institutional or federated datasets that encompass a diverse range of healthcare systems and populations. This approach would help ensure that models trained on EHR data are more representative and adaptable to varying clinical environments. Additionally, strategies such as external validation on datasets from other institutions, geographic regions, or healthcare systems could further enhance the robustness and applicability of the results.

Additionally, a significant barrier arises from the insufficient training provided to staff, which hinders their ability to utilize these advanced technologies to their full potential [102,103]. Training healthcare staff is crucial to the successful implementation of AI in AMS. However, many healthcare professionals do not receive adequate training on using AI technologies or best practices for prescribing antimicrobials. A lack of regular educational programs can lead to a poor understanding of prescription guidelines and the proper use of antibiotics [104].

**Figure 3:**
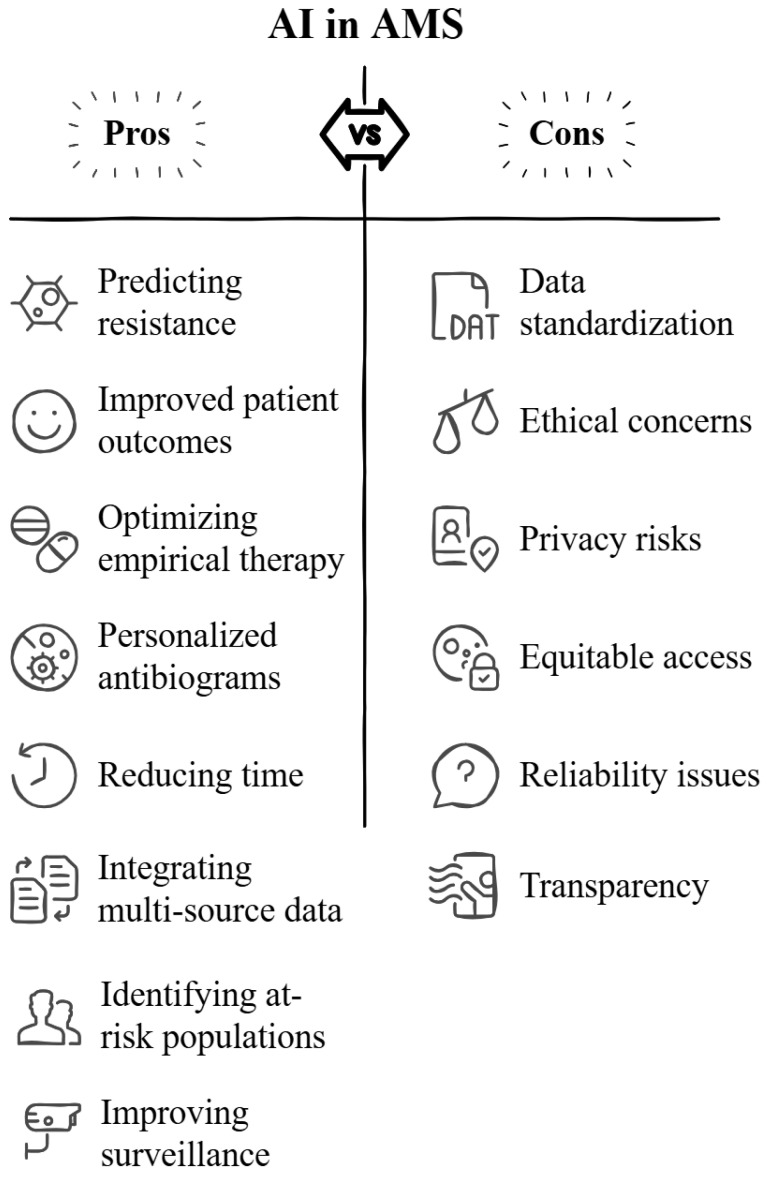
Overview of the advantages and challenges associated with using artificial intelligence (AI) in antimicrobial stewardship (AMS), highlighting its potential for improving resistance prediction, patient outcomes, and surveillance, while addressing ethical and reliability concerns.

Figure 3 summarizes the main advantages and limitations of AI integration in AMS, providing a balanced overview of its potential and challenges.

## 6. Priorities for AI Integration in Antimicrobial Stewardship

To effectively address the complexities of integrating artificial intelligence (AI) into antimicrobial stewardship (AMS), it is crucial to establish a set of clear and definitive research priorities. First and foremost, the development of comprehensive guidelines for the application of AI in clinical environments is absolutely essential. These guidelines should cover critical aspects such as privacy protection and the mitigation of bias, ensuring that data management practices are responsible and ethical.

In addition to these guidelines, there is a pressing need for the implementation of rigorous data quality standards and robust sharing protocols. This step is vital, as it ensures that the AI models are trained on data that is both accurate and representative, which in turn helps to eliminate disparities that can arise across diverse populations.

Moreover, we must focus on creating AI models that are adaptable to various healthcare contexts, taking into account the local variations in antimicrobial resistance. This adaptability will enable healthcare providers to tailor interventions more effectively to the specific challenges faced in their communities.

Another critical area for research is the conduct of longitudinal studies that investigate the long-term effects of AI on public health and clinical practices. The absence of longitudinal data poses a significant limitation in evaluating the sustained impact of these tools. Short-term studies provide valuable insights into initial model performance, such as predictive accuracy or diagnostic efficiency, but they may not capture critical aspects of long-term efficacy, including the durability of model predictions, adaptability to evolving resistance patterns, and the overall influence on antimicrobial resistance trends. Longitudinal research endeavors are essential, as they will yield concrete evidence about AI’s effectiveness in improving AMS and overall health outcomes. For example, resistance patterns and prescribing practices often shift due to changing microbial epidemiology, healthcare policies, and patient demographics. Without longitudinal data, it is challenging to determine whether AI-driven interventions can adapt to these changes or if their benefits diminish over time. Moreover, the long-term consequences of using AI in AMS—such as the reduction in broad-spectrum antibiotic use, prevention of resistance emergence, and improvement in patient outcomes—require sustained observation to be fully understood. Future research should prioritize the design and implementation of longitudinal studies that assess the performance, safety, and adaptability of AI and ML models over extended periods. Such studies could also explore the cost-effectiveness of these tools and their impact on healthcare infrastructure, providing critical evidence to guide policy decisions and promote widespread adoption.

By proactively addressing these multifaceted challenges, we can significantly enhance the role of AI in the global fight against antimicrobial resistance, ultimately leading to better health outcomes for populations around the world (Table 1).

## 7. Materials and Methods

A comprehensive review was conducted to explore the role of AI and ML in AMS and combating AMR. Searches were systematically performed across PubMed/MEDLINE, Scopus, EMBASE, and Web of Science (WoS) for articles published in English, similar to our previous studies [105,106]. The search strategy employed a combination of keywords and Medical Subject Headings (MeSH) terms, including “artificial intelligence”, “machine learning”, “antimicrobial stewardship”, and “antimicrobial resistance”, using Boolean operators “AND” and “OR” to optimize the retrieval of relevant studies. Eligible articles were selected based on their focus on AI/ML applications in AMS, resistance prediction, or diagnostic advancements. Particular emphasis was placed on evaluating the implications of AI/ML for public health, ethical aspects such as data privacy and algorithmic bias, and inherent challenges associated with their integration into AMS, including issues related to data quality, algorithm transparency, and scalability. This comprehensive approach provided an extensive understanding of AI’s transformative role in AMS, highlighting both its potential benefits and critical areas for further research and development. 

## 8. Conclusions

AI and ML have emerged as transformative tools in addressing the global challenge of AMR, offering unprecedented capabilities in prediction, diagnostics, and public health strategies. By integrating diverse and complex datasets, these technologies enhance the precision of AMS programs, streamline diagnostics, and optimize resource allocation. However, the successful implementation of AI/ML in AMS is not without challenges, including issues of interpretability, data quality, algorithmic bias, and logistical barriers in real-world integration.

To fully realize the potential of AI and ML in AMS, future efforts must prioritize the development of transparent and interpretable models, foster interdisciplinary collaborations between data scientists and clinicians, and address ethical considerations such as data privacy and equitable access. Moreover, expanding research to include diverse settings, particularly low-resource environments, will be crucial for ensuring global applicability and fairness. The integration of these technologies into AMS represents a paradigm shift, aligning with the broader goals of public health to combat AMR effectively and sustainably. By addressing the existing barriers and building on the insights discussed in this review, AI and ML hold the promise of transforming AMS into a more precise, efficient, and equitable endeavor, safeguarding the efficacy of antibiotics for future generations.

## Figures and Tables

**Table 1:** Summary of the key challenges and corresponding mitigation strategies.

Challenge	Mitigation Strategy
Privacy concerns related to sensitive patient data, risk of discrimination, and bias in AI models due to non-representative datasets.	Develop comprehensive guidelines addressing privacy and bias; establish responsible data management practices and protocols.
Poor data quality, inconsistency in microbiological data, and difficulties in validating AI models in real-world clinical settings.	Implement rigorous data quality standards, conduct randomized clinical trials, and create adaptable AI models for local contexts.
Lack of leadership support, inadequate resources, outdated infrastructure, poor integration with EHRs, and insufficient staff training.	Secure organizational support, modernize infrastructure, enhance EHR integration, and provide comprehensive staff training.
Inequitable performance across demographic groups, perpetuating disparities in healthcare.	Train AI models with diverse datasets; ensure inclusivity during algorithm design and validation processes.
Difficulty in seamlessly integrating AI technologies into the current healthcare systems and practices.	Design AI tools compatible with existing systems and workflows; provide user-friendly interfaces for easier adoption.
Limited evidence of AI’s effectiveness in real-world AMS scenarios over time.	Conduct longitudinal studies to assess AI’s impact on public health and clinical practices.

## Data Availability

No new data were created or analyzed in this study.
